# Supplementary material for: Investigation of the status of rest facilities at industrial sites and awareness of relevant laws and regulations of South Korea
Source: PLoS One. 2024 Jun 7;19(6):e0302654. doi: 10.1371/journal.pone.0302654 (PMC11161079; doi:10.1371/journal.pone.0302654)
Supplement: S1 Questionnaire — (DOCX) [file pone.0302654.s001.docx]

Article 128-2 (1) of the Occupational Safety and Health Act Regarding the installation of rest facilities

| ※ Thank you for giving us your valuable time during your busy schedule.  My name is OOO, and I am a doctoral student at Seoul National University of Science and Technology.  This questionnaire was administered on 22.08.18. In relation to the mandatory installation of rest facilities in Article 128-2 (1) of the Occupational Safety and Health Act,  This is a survey for research purposes to understand the current status of rest facility installations at workplaces and devise improvement measures.  The survey contains a total of 37 questions. The responses will not be used for any purpose other than research purposes, and will be safely disposed of after being stored for 3 years at the end of the research period..  Although there are no direct compensation benefits to survey participants, we would like to help improve the working environment. If you have any questions when filling out the survey, please contact us below. Please note that you can stop the survey at any time if you wish to do so. |
| --- |

1. What is your gender? 🞎 Man 🞎 Woman
2. What is your age range? 🞎 20~29 🞎 30~39 🞎 40~49 🞎 Over 50
3. What industry do you work in?

🞎 Manufacturing business 🞎 Construction industry 🞎 Service industry

1. In the case of other service industries, what occupations are applicable?

🞎 telephone counselor 🞎 care service worker 🞎 telemarketer

🞎 delivery man 🞎 cleaner 🞎 apartment security guard

🞎 building security guard 🞎 etc

1. Are you a manager or a worker?

🞎 Manager 🞎 Worker

1. What is the size of the business?

🞎 <20 peaple 🞎 20~50 peaple 🞎 51~300 peaple 🞎 >300 peaple

1. Are rest facilities installed? 🞎 Yes 🞎 No
2. Why not installed the rest facility? 🞎 Yes 🞎 No
3. Is it segregated into male and female? 🞎 Yes 🞎 No
4. Is it more than 6square meters? 🞎 Yes 🞎 No
5. Is the area suitable for simultaneous use? 🞎 Yes 🞎 No
6. Are they taller than 2.1 meters? 🞎 Yes 🞎 No
7. Is there air conditioning/heating? 🞎 Yes 🞎 No
8. Why is there no air conditioning?

🞎 Problems with installation location of rest facilities (e.g. electrical work, etc.)

🞎 business owner's consciousness

🞎 cost issue

🞎 Due to lack of worker interest or ignorance of the law

🞎 etc.

1. Is it in a safe place from fire and explosion hazard? 🞎 Yes 🞎 No
2. Why is it installed in a fire and explosion hazard area?( If you check “No” in Q15)

🞎 Problems with installation location of rest facilities (e.g. electrical work, etc.)

🞎 business owner's consciousness

🞎 cost issue

🞎 Due to lack of worker interest or ignorance of the law

🞎 etc.

1. Is it installed in a safe place from noise and dust? 🞎 Yes 🞎 No
2. Why is it installed in a noise and dust?( If you check “No” in Q17)

🞎 Problems with installation location of rest facilities (e.g. electrical work, etc.)

🞎 business owner's consciousness

🞎 cost issue

🞎 Due to lack of worker interest or ignorance of the law

🞎 etc.

1. Is water provided? 🞎 Yes 🞎 No
2. Are there chairs? 🞎 Yes 🞎 No
3. Is there a rest facility manager? 🞎 Yes 🞎 No
4. Are you using the rest facilities? 🞎 Yes 🞎 No
5. Why not use the rest facility? ( If you check “No” in Q22)

🞎 Unnecessary

🞎 Because the rest facilities are not comfortable

🞎 Because the space is small

🞎 Because the manager noticed

🞎 Inconvenient because it is not a dedicated rest area

🞎 Rest facilities are far away

🞎 etc.

1. Is it a private resting facility? 🞎 Yes 🞎 No
2. Are you maintaining the right temperature? 🞎 Yes 🞎 No
3. Are you maintaining proper humidity? 🞎 Yes 🞎 No
4. Is the lighting adequate? 🞎 Yes 🞎 No
5. Are you satisfied with the currently installed rest facilities?

🞎 very much yes 🞎 yes 🞎 nomal 🞎 no 🞎 very much no

1. Do you know that the rest facility installation law was enacted?

🞎 very much yes 🞎 yes 🞎 nomal 🞎 no 🞎 very much no

1. Do you think rest facility manager is needed?

🞎 very much yes 🞎 yes 🞎 nomal 🞎 no 🞎 very much no

1. Do you think rest facilities are essential?

🞎 very much yes 🞎 yes 🞎 nomal 🞎 no 🞎 very much no

1. Do you think rest facilities are helpful in preventing safty accidents?

🞎 very much yes 🞎 yes 🞎 nomal 🞎 no 🞎 very much no

1. Do you think rest facilities are helpful for improving health?

🞎 very much yes 🞎 yes 🞎 nomal 🞎 no 🞎 very much no

1. Do you think a business with less than 20 employees needs financial support?

🞎 very much yes 🞎 yes 🞎 nomal 🞎 no 🞎 very much no

1. Do you think a penalty of 15million won for not installing rest facilities is appropriate?

🞎 very much yes 🞎 yes 🞎 nomal 🞎 no 🞎 very much no

1. Do you think a penalty of 5million won is appropriate when the rest facility is below the standard?

🞎 very much yes 🞎 yes 🞎 nomal 🞎 no 🞎 very much no

1. Do you think the enactment of the law will help improve rest facilities?

🞎 very much yes 🞎 yes 🞎 nomal 🞎 no 🞎 very much no

※ Survey has ended. Thank you for your effort (By clicking the submit button below, you agree to the collection of personal information and survey responses and complete submission.)
