# Supplementary material for: Investigation of the status of rest facilities at industrial sites and awareness of relevant laws and regulations of South Korea
Source: PLoS One. 2024 Jun 7;19(6):e0302654. doi: 10.1371/journal.pone.0302654 (PMC11161079; doi:10.1371/journal.pone.0302654)
Supplement: Attachment — Submitted filename: response to reviewers.docx [file pone.0302654.s002.docx]

- Response to reviewer's #1 comments on revision

The revised texts are marked in red on the manuscript.

1. Add the full term for OECD and use an abbreviated one (OECD) hereafter.

**Organization for Economic Cooperation and Development was added in the third line of the introduction.**

2. The sentence in the 18th line of the introduction is incomplete, so please rewrite it.

We revised the sentence in line 18 of the introduction completely. Please refer to the sentence.

3. Consider deleting the content stating that factors such as dining facilities, changing rooms, and rest areas account for 60.9% of the satisfaction level in construction sites in the research section of the introduction or repositioning it with the positive aspects of rest.

We revised to make it smooth by relocating the sentence 'Research on the satisfaction ~ ' in line 19 of the introduction into the beginning of the previous study of Son, Chang-Baek.

4. Provide a detailed explanation of the methodology and add specific details regarding the validity and reliability of the survey items

We added the following texts to the method section, detailing the recruitment of subjects and selection of survey items. The reliability analysis results that a Cronbach's α was 0.781, indicating a high reliability had already been mentioned.

- The study subjects were recruited after obtaining consent to participate in the survey anonymously among working people (excluding minors).
- Questionnaire items were created based on the detailed items in the standards for installing and managing rest facilities under Article 194-2 of the Enforcement Regulations of the Occupational Safety And Health Act (see table 1).

5. Make sure to clarify any unclear aspects regarding the even distribution of survey subjects across different age groups.

[The distribution across age groups from the 20s to the 50s was relatively even, ranging from 15.4% to 38.8%. However, only one person, accounting for 0.4%, was over the age of 60.]

The above content was revised as follows.

- Looking at the participants in the survey by age group, those in their 20s accounted for 15.4%, those in their 30s accounted for 24.7%, those in their 40s accounted for 38.8%, and those aged 50 and older accounted for 21.1%. (see Table 2)

6. The survey method section did not mention the reasons for not installing rest facilities.

The following content was added to the method section.

- For items related to the reasons for not installing rest facilities (Q2-1, Q2-2), the options were selected based on interviews with on-site workers and managers, and the most common opinions were lack of space, cost related issues, lack of awareness by employers, and lack of knowledge about legal matters.

7. There is insufficient discussion regarding the reasons for the lack of rest facilities in the line 24, and the focus seems to be solely on musculoskeletal disorders.

We added the texts that no prior research regarding the reasons for not installing rest facilities has been found since the mandatory legislation for rest facility installation took effect in Korea on August 18, 2022. Additionally, all content related to the necessity of rest facilities with a focus on musculoskeletal disorders has been removed.

8. In the introduction, we mentioned the laws related to rest facilities in Turkey, the United Kingdom, India, and Australia, but there is no comparison in the discussion with Korea.

We added relevant content to the discussion section. Please refer to it.

9. The conclusion should clearly summarize the findings and key points. It is necessary to emphasize the importance of existing literature and recommend future research directions.

The ending part of the conclusion was revised, taking into account the reviewer's comments.

- Response to reviewer's #2 comments on revision

1. Add the full term for OECD and use an abbreviated one (OECD) hereafter.

**Organization for Economic Cooperation and Development was added in the third line of the introduction.**

2. Provide a detailed explanation of the methodology and add specific details regarding the validity and reliability of the survey items.

We added the following texts to the method section, detailing the recruitment of subjects and selection of survey items. The reliability analysis results that a Cronbach's α was 0.781, indicating a high reliability had already been mentioned.

- The study subjects were recruited after obtaining consent to participate in the survey anonymously among working people (excluding minors).
- Questionnaire items were created based on the detailed items in the standards for installing and managing rest facilities under Article 194-2 of the Enforcement Regulations of the Occupational Safety And Health Act (see table 1).

3. The survey method section did not mention the reasons for not installing rest facilities.

The following content was added to the method section.

- For items related to the reasons for not installing rest facilities (Q2-1, Q2-2), the options were selected based on interviews with on-site workers and managers, and the most common opinions were lack of space, cost related issues, lack of awareness by employers, and lack of knowledge about legal matters.

4. Please explain Korea’s labor laws in comparison to other countries.

A discussion was made by comparing the laws related to rest facilities in Turkey, the UK, India, and Australia mentioned in the introduction with the laws related to rest facilities in Korea.

5. The conclusion should clearly summarize the findings and key points. It is necessary to emphasize the importance of existing literature and recommend future research directions.

The ending part of the conclusion was revised, taking into account the reviewer's comments.
